# Whole-Genome Sequencing of a Vibrio cholerae El Tor Strain Isolated in the Imported Cholera Focus in Siberia

**DOI:** 10.1128/genomeA.01550-14

**Published:** 2015-03-26

**Authors:** S. V. Balakhonov, L. V. Mironova, E. A. Basov, A. S. Gladkikh, M. V. Afanasev, V. S. Ganin, L. Y. Urbanovich, E. A. Sidorova

**Affiliations:** Irkutsk Antiplague Research Institute, Irkutsk, Russia

## Abstract

The draft genome sequence of Vibrio cholerae O1 strain I-1263, isolated from a patient in the imported focus in Siberia, was determined. The established structural features of the mobile genetic elements indicate stage-by-stage formation of a highly pathogenic V. cholerae clone and promote understanding of the mechanisms of evolutionary pathogen transformations.

## GENOME ANNOUNCEMENT

Atypical variants of Vibrio cholerae El Tor appeared in the beginning of the 1990s and contained a cholera toxin subunit B gene of the classical type (*ctxB1*) (1) that caused all of the epidemic cholera complications registered during that period in Siberia and the Far East (2). Analysis of the genetic structure of atypical V. cholerae El Tor variants isolated in Siberia and the Far East in the 1990s demonstrated its differentiation to some genotypes (3). The V. cholerae El Tor O1 I-1263 strain isolated in the imported focus in Irkutsk city in 1997 from a patient followed from Uzbekistan was attributed to one of these genotypes (III b). Analysis of CTX prophage structure revealed both the presence of nucleotide replacements in the *ctxB* gene (C/T in the 115 and 203 positions) and also a smaller number of repeats in the P_ctxAB_ promoter region (3). In addition, detection of VSP-I and VSP-II in PCR showed deficiency of the pro496 locus VSP-II in this strain.

Deciphering of the nucleotide sequence of V. cholerae El Tor O1 I-1263 genome was performed for profound analysis of its genetic organization and for further phylogenetic analysis.

Genome libraries were constructed according to Roche’s protocol with the reagents of the GS Junior Titanium series. Sequencing was implemented using Roche GS Junior. Primary processing of reads was performed using GS Reference software v. 2.8 by alignment on the genome of the V. cholerae El Tor O1 N16961 reference strain. The draft genome of the V. cholerae El Tor O1 I-1263 strain included 50 contigs, with a total length of 3,957,767 bp. The average length of a contig was 80,764 bp, with a maximal contig length of 656,754 bp and an *N*_50_ of 236,680 bp. The percentage of mapped reads was 96.7%. An average covering of the genome was 13, with maximal coverage of 41. Genome annotation was achieved using the RAST server. A total 3,985 protein-coding and 123 RNA-coding sequences were revealed. The draft genome revealed 29 genes associated with phages, prophages, and mobile elements, 7 additional genes of pathogenicity, and 60 genes providing resistance to antibiotics.

The analysis of the CTX prophage region revealed high homology with V. cholerae El Tor O1 N16961 genes. Two nucleotide replacements in the 115/203 positions were identified in the *ctxB* gene sequence. The presence of three tandem repeats (5-TTTTGAT-3) was established in the structure of the P_ctxAB_ promoter area, which conformed to our earlier results (3). Deletion of the VC0496-to-VC0498 loci was revealed in the VSPII region. According to Taviani et al., the strains that appeared after 2004 with extended VSPII deletions covering the VC0496-to-VC0512 loci were characterized by increased epidemic potential, and in previous years these variants displaced earlier circulating atypical V. cholerae El Tor strains with intact VSPII (4). The tested V. cholerae El Tor O1 I-1263 strain with deletion of three VSPII loci may be an intermediate in the formation of a new clone of the highly pathogenic V. cholerae El Tor variant.

### Nucleotide sequence accession number.

The whole-genome shotgun project for Vibrio cholerae O1 El Tor strain I-1263 has been deposited at DDBJ/EMBL/GenBank under the accession no. JPLT00000000.

## References

[1] Safa A, Nair GB, Kong RY 2010 Evolution of new variants of *Vibrio cholerae* O1. Trends Microbiol 18:46–54. doi:10.1016/j.tim.2009.10.003. 19942436

[2] Mironova LV, Balakhonov SV, Urbanovich L, Polovinkina VS, Kozhevnikova AS, Kulikalova ES, Afanasev MV 2011 Detection of “hybrid” *Vibrio choleraе* El Tor strains at epidemic complications in Siberia and at the Far East. Zh Mikrobiol Epidemiol Immunobiol 5:12–18. (In Russian.) 22145342

[3] Mironova LV, Balakhonov SV, Urbanovich LY, Kozhevnikova AS, Polovinkina VS, Kulikalova ES, Afanasiev MV 2012 Molecular-genetic analysis of *Vibrio cholerae* El Tor strains of epidemic risk isolated in Siberian and Far East regions of Russia. Mol Genet Microbiol Virol 27:61–68. doi:10.3103/S0891416812020073. 22937565

[4] Taviani E, Grim CJ, Choi J, Chun J, Haley B, Hasan NA, Huq A, Colwell RR 2010 Discovery of novel *Vibrio cholerae* VSP-II genomic islands using comparative genomic analysis. FEMS Microbiol Lett 308:130–137. doi:10.1111/j.1574-6968.2010.02008.x. 20528940 PMC2925232

